# Influence of Interfacial Interaction and Composition on Fracture Toughness and Impact Properties of Carbon Fiber-Reinforced Polyethersulfone

**DOI:** 10.3390/polym16060860

**Published:** 2024-03-21

**Authors:** Valerii G. Torokhov, Dilyus I. Chukov, Victor V. Tcherdyntsev, Andrey A. Stepashkin, Mikhail Y. Zadorozhnyy

**Affiliations:** 1 Laboratory of Functional Polymer Materials, National University of Science and Technology “MISIS”, Leninskii Prosp, 4, 119049 Moscow, Russia; vgtorohov@gmail.com (V.G.T.); dil_chukov@mail.ru (D.I.C.); a.stepashkin@misis.ru (A.A.S.); zadoroznyy.m.yu@mail.ru (M.Y.Z.); 2 Center for Project Activities, Moscow Polytechnic University, Bolshaya Semenovskaya Str., 2, 107023 Moscow, Russia

**Keywords:** carbon fibers, polyethersulfone, thermoplastic composites, surface modification, fracture toughness, impact strength, mechanical properties

## Abstract

In this study, the interlaminar fracture toughness and impact strength of polyethersulfone reinforced with continuous carbon fibers were studied. Interlaminar fracture toughness tests were performed using the double cantilever beam method. It was shown that surface modification using the thermal oxidation method of the carbon fibers can strongly increase the interlaminar fracture toughness of the obtained composites. Thus, the maximum value reached 1.72 kJ/m^2^, which was 40% higher than the fracture toughness of the composites reinforced with initial carbon fibers. Moreover, fractographic analysis using a scanning electron microscope allowed us to highlight the main reasons for the dependence of fracture toughness on fiber content and surface modification conditions of the carbon fibers. It was shown that the main factor that allowed for an increase in fracture toughness was the enhanced interfacial interaction between the fibers and polymer matrix. Additionally, it was found that expectedly, there was a good correlation between interlaminar fracture toughness and interlaminar shear strength results. However, a negative influence of surface modification on the impact properties of composites was found. Such behavior occurred because of higher structural stability and lower exposure to delamination in multiple layers of the composites reinforced with the modified carbon fibers. It was found that impact energy reached ~150 kJ/m^2^ for the polyethersulfone-based composites reinforced with initial fibers, while the composites reinforced with modified carbon fibers showed impact energy values of only ~80 kJ/m^2^. Nevertheless, surface modification of carbon fibers using the thermal oxidation method can be an effective method for improving the performance properties of polyethersulfone-based composite materials.

## 1. Introduction

Carbon fiber-reinforced polymers (CFRPs) are highly demanded materials, used in aerospace, automobile, sport, and other industries. Combining high mechanical properties with low weight, these composites have become one of the most developed and studied materials in the world [1,2,3]. However, existing CFRPs are commonly made of epoxy resin, a well-known thermoset polymer. The thermoset nature explains the existing drawbacks of this class of composite materials, which include bad recyclability and repairability, difficulties with automatization processes of impregnation and formation, a limited live gap of prepregs, etc. Moreover, thermoset CFRPs tend to have worse impact properties, compared with composites based on thermoplastic matrices, while the fracture resistance of thermoset polymers is usually lower than that of thermoplastics [4]. Apart from that, in multiple studies, polyethersulfone (PES) was added into uncured epoxy resin to increase the fracture toughness of composites [5,6]. Chao et al. reported an increase in interlaminar fracture toughness up to 61.5% after the introduction of polyhersulfone films in interlaminar areas of epoxy/CF composite systems [5]. Noteworthy, the increase in the thickness of PES films led to better fracture resistance. Multiple authors investigated the fatigue properties of carbon fiber-reinforced polyetheretherketone, which is a thermoplastic polymer, and reported that it tends to have higher fatigue properties compared with a CFRP with an epoxy resin matrix [7,8].

Consequently, in recent years, the focus of researchers moved to CFRPs based on thermoplastic polymers. Their potential for production automation, as well as recycling capability, intensified the existing advantages of CFRPs [9,10]. Moreover, CFRPs based on thermoplastics tend to have better interlaminar fracture toughness and impact strength due to less brittleness compared with epoxy resin [11]. Among the most significant polymer matrices for such composites are polyphenylene sulfide (PPS), polyetheretherketone (PEEK), polycarbonate (PC), polysulfone (PSU), polyethersulfone (PES), and others [12]. In our previous studies, the mechanical and thermophysical properties of CFRPs based on PSU and PES were studied [13,14,15,16]. It was shown that surface modification of carbon fibers using the thermal oxidation method in an ambient atmosphere significantly increases some properties of composites reinforced with modified fibers, such as flexural and tensile strength, interlaminar shear strength, and heat deflection temperature. That behavior occurred as a result of the formation of new functional groups on the surface of the CF and decomposition of a sizing on the CF surface, which results in improved interfacial interaction between the fibers and polymer matrix. It was noteworthy that the full decomposition of sizing appeared only at a temperature of 500 °C, while at lower temperatures of heat treatment, only partial oxidation was observed.

However, while the tensile and flexural strengths of CFRPs are uniquely high, their impact strength and fracture toughness are considered weak points, especially for thermoset-based composites, making them relevant and important to research. Since the fracture in carbon fiber-reinforced composites distributes mostly through the interlaminar area, delamination by interlaminar fracture should be considered as a prevalent mechanism of fracturing [17]. Consequently, the increase in the interlaminar fracture toughness (ILFT) of composites is a relevant challenge, especially demanded in areas where the material is exposed to impacts of different energy since such impacts strongly influence the intra- and interlaminar damage mechanisms of the composites [18]. Moreover, an increase in fracture toughness may be further evidence of an enhanced interfacial interaction between the fibers and polymer matrix [17,19].

In the scientific literature, some general ways of enhancing ILFT are reported. One of the promising methods is introducing different fillers, especially nanomaterials, such as carbon nanotubes and other fillers, into a polymer matrix, which can significantly increase the ILFT of composites, as reported in [20,21]. The incorporation of an additional phase in a matrix also allows for physically delaying the propagation of a crack. Solodilov V. I. et al. [22] reported that introducing PES powder into an epoxy resin-based CFRP significantly increases mode I interlaminar fracture toughness. However, this method has a drawback, reflected in a noticeable increase in the viscosity of a polymer matrix [23]. Such a method was implemented for thermoplastic CFRPs as well. Wang X. et al. [24] described the mechanism of drastic improvement in the interlaminar fracture toughness of PEEK/CF composites by introducing carbon nanotube-doped PEEK films between layers of composites. It was shown that the observed behavior was due to crack path deflection caused by CNT. Song J. et al. [25] also modified CF/PEEK composites with CNT and showed that the mode I and II interlaminar fracture energies of the composites with 0.5 wt.% CNTs increases to 78.8% and 90.5% to neat CF/PEEK composites. Another approach is the interlaying of different non-woven polymer fibers in adjacent plies, which allows for increasing ILFT by reducing the brittleness of a polymer matrix and bridging the polymer fibers [23,26,27]. Surface modification of reinforcing fibers can also be a way to enhance interlaminar fracture toughness. Downey M. A. stated that treatment of carbon fibers by ultra-violet and ozone resulted in improved fracture properties of micro-composites [28].

The mechanical behavior of composites during impact is another important issue to research. Impact strength is especially demanded in the automobile industry, where the body of a car must absorb as much energy during an impact as possible. Introducing rubber in a matrix of a composite is considered the most effective way to increase the impact strength of polymers as well as CFRPs [29,30]. The existing drawback of lowering the quasistatic mechanical properties of composites forces researchers to develop alternative ways of increasing impact strength. Thus, Kim et al. [30] investigated the influence of different surface modifications of CF on the impact properties of composites reinforced with short CF and rubber. It was shown that the impact strength of the composites was higher compared with pure polymer matrix but enhancing the interfacial bonding between the CF and matrix by various surface modification methods resulted in lower values of impact strength. This phenomenon was explained by the acceleration of the formation of delaminated zones in the composite by the improved interfacial bonding in the region of stress concentration, resulting in more notch-sensitive materials. Cheon J. and Kim M. [31] also showed an inverse relationship between ILSS and impact strength values. The ILSS of CNT-mixed CF-reinforced polyamide-6 was reduced from 35 MPa to 28 MPa by mixing the CNT, while the Izod impact resistance of the composites was increased by 24% from 191 kJ/m^2^ to 231 kJ/m^2^ with an MWCNT concentration of 3 or 5 wt.%. due to absorbing the impact energy by delamination, matrix cracking, fiber breakage, and fiber pull-out, which were more pronounced in the case of lower interfacial interaction.

Generally, only a few papers concerning the introduction of PES films, powders, or nanofibers are available to the public [5,6,24]. It was reported that adding polyethersulfone into epoxy/CF systems allowed for significantly increasing interlaminar fracture toughness of composites. However, there are no studies available concerning PES/CF composite systems, especially their fracture and impact properties. Moreover, the influence of the thermal treatment of carbon fibers on the delamination of CFRPs has not been studied widely. Considering the present studies showing that the surface modification of carbon fibers can increase some mechanical properties of CFRPs based on PES, while fiber content also has a huge influence on the properties of materials, it can be predicted that the same behavior will occur in terms of fracture toughness and impact strength. Thus, this paper is aimed at studying the influence of carbon fiber content and thermal treatment conditions of fibers on the fracture toughness and impact strength of carbon fiber-reinforced polyethersulfone-based composites.

## 2. Materials and Methods

To investigate the interlaminar fracture toughness and impact strength of the carbon fiber-reinforced polyethersulfone-based composites, experimental samples were obtained using a solution-based technique, developed and thoughtfully described in our previous research [16]. Following this method, twill-weaved carbon fabric 3K-1200-200 (“HC Composite” JSC, Moscow, Russia) was impregnated by 20 wt.% polyethersulfone Ultrason E2020 P SR Micro (BASF, Ludwigshafen, Germany) solution in N-2-methylpyrrolidone (Eastchem, Changzhou, China) in different proportions. After drying the impregnated fabrics, prepregs with fiber contents of 50, 60, and 70 wt.% were formed. The resulting prepregs were cut and compression-molded at a temperature of 350 °C and a pressure of 10 MPa for 30 min in an air atmosphere. Hereinafter, the composites reinforced with initial fibers with fiber contents of 50, 60, and 70 wt.% are marked as 50/50, 60/40, and 70/30, respectively. Since composites with a fiber content of 50 wt.% showed the best performance in terms of mechanical properties, as described in [16], modified CF-reinforced composites were obtained with that fiber content. Surface modification of the carbon fibers was conducted using thermal oxidation in an air atmosphere at temperatures of 300 and 500 °C for 30 min. Therefore, the composites reinforced with modified fibers with fiber contents of 50 wt.% are marked as TO 300 and TO 500, respectively.

**Figure 1:**
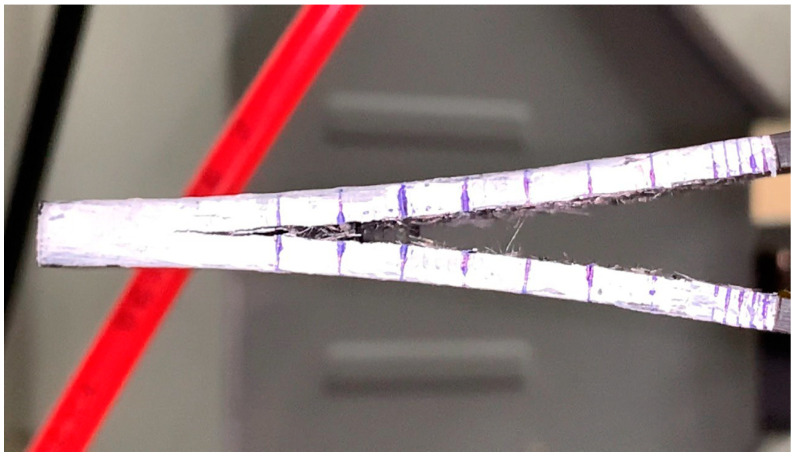
Typical view of a carbon fiber-reinforced PES-based composite specimen during ILFT tests.

Interlaminar fracture toughness test was conducted on a Zwick/Roell Z020 (Zwick GmbH & Co., Ulm, Germany) universal test machine in consideration of the ASTM D5528 standard [32], which allowed for determining mode I fracture toughness. Despite the fact that this standard applies to unidirectional composites, it was also used for investigating the ILFT of composites with different geometries of reinforcing materials [33]. For this purpose, samples with a length of 110 mm, a width of ~22 mm, and a thickness of 5 mm were obtained. Non-adhesive polyimide film with a thickness of 40 µm was used as an initial crack, which was introduced at the midplane to a length of ~50 mm. Piano hinges were used as joints between a sample and the testing machine. To facilitate the monitoring of the propagation of a crack during the experiment, the observed side of a sample was painted white and marked. First, 5 marks were put with at an interval of 1 mm, and all the following marks lied after 5 mm. Figure 1 illustrates a sample during the experiment. All the samples were precracked with the testing machine before experiment.

During the experiment load–displacement curves were obtained, and the propagation of a crack was recorded, which allowed for correlating a load–displacement curve with the length of a crack. According to the ASTM D5528 standard, a fracture energy G_1C_ was determined with regard to modified beam theory with the formula:

(1) $$G_{1C}=\frac{3\delta P}{2ba},$$

where P is the applied load (N), δ is the displacement (mm), b is the width of a sample (mm). and a is the length of a crack (mm).

**Figure 2:**
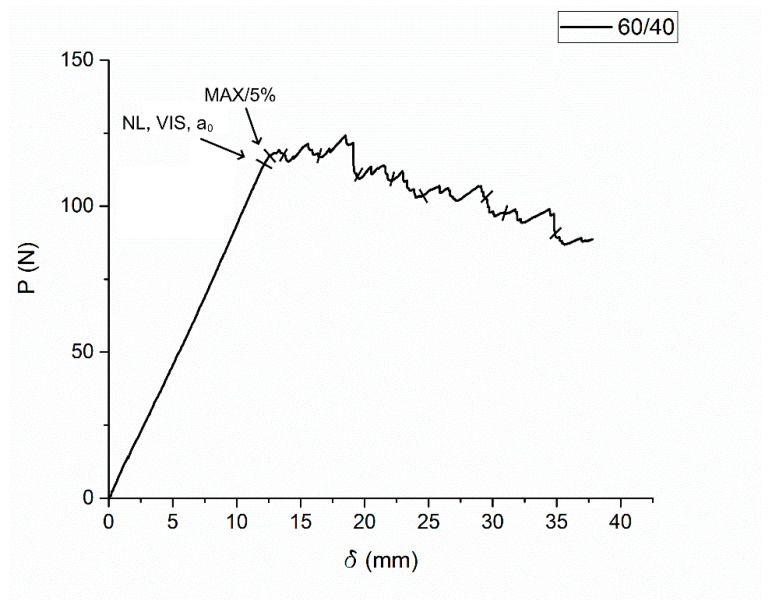
Typical load–displacement curve (60/40 composites, our study) during ILFT test.

There are multiple ways to determine the start of delamination [34]. The first one is to determine a point from which the load–displacement curve starts to be non-linear. An assumption occurs when determining the point of non-linearity, according to which a crack starts to propagate inside the sample, while the fracture is not visible on the side plane (NL point). The second is to find a point from which a fracture starts to be visible on a side plane (VIS point). In the case of a brittle matrix, these two points can be the same. The third way is to determine a point on a load–displacement curve that corresponds to the intersection between the curve and a straight line with a slope 5% smaller than the slope of the load–displacement curve before the point of non-linearity (5% point). If the intersection occurs later than the maximum point, this maximum point is considered as wanted (MAX point). All three points are shown in Figure 2.

Modified beam theory was chosen as a method to analyze the data as it gives the most conservative values of G_1C_ and requires the least amount of calculation. However, this method requires a data reduction procedure that allows for correcting the value of G_1C_, obtained with Formula (1), which is typically overestimated because of crack tip rotation. To correct the results, the length of a crack is considered to be slightly more than the real length of a crack on a value. Thus, fracture energy can be found by the formula:

(2) $$G_{1C}=\frac{3\delta P}{2b(a+∆)},$$

**Figure 3:**
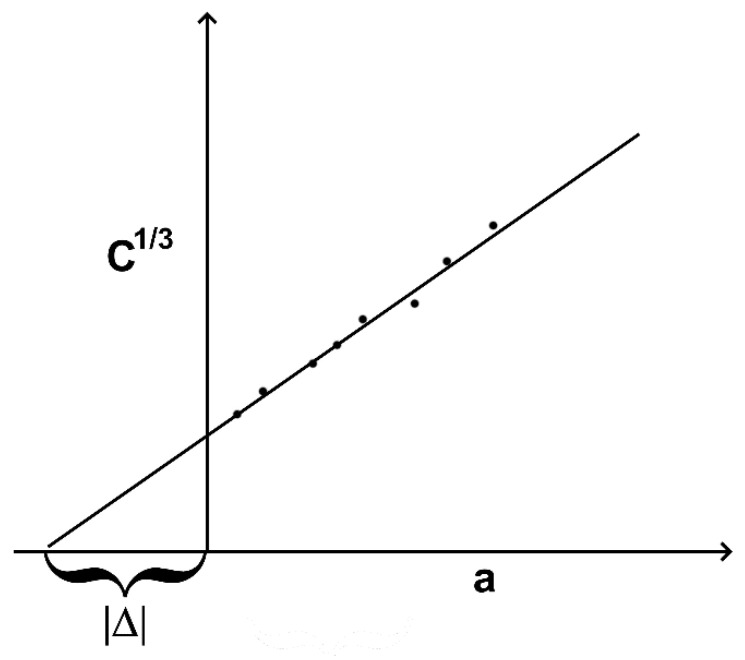
Principal scheme of obtaining a correction value Δ.

The value of Δ can be found by generating a plot of cubic root of compliance C, which is considered δ/P, as a function of length of a crack a. Thus, Δ can be found as a negative line segment, cut off by extrapolating to horizontal axis plot and vertical axis. A scheme showing how to obtain a Δ value is provided in Figure 3.

Moreover, after finding Δ, the effective E-modulus during flexural deformation can be found via the formula:

(3) $$E_{1f}=\frac{64{\left(a+∆\right)}^{3}P}{\delta bh^{3}},$$

Thus, a comparison between the E-modulus, obtained during flexural strength tests, and the value, calculated with Formula (3), can be considered as an indicator of the correctness of the applicability of the assumptions made in modified beam theory.

For a better understanding of delamination processes, scanning electron microscopy via a TESCAN VEGA COMPACT microscope (TESCANORSAYHOLDING, a.s., Brno-Kohoutovice, Czech Republic) was conducted. Since the investigated materials have low electrical conductivity and charge by electron beam, the samples were coated with platinum with a thickness of ~10 nm.

Impact strength tests were conducted using a Zwick 5113 pendulum impact tester (Zwick GmbH & Co, Ulm, Germany). The test was conducted with the Sharpy scheme according to the ASTM D 5942-96 [35] standard. The kinetic energy of the pendulum was 15 J. For this test, samples with a thickness of 55 mm, a width of 10, and a length of 110 mm were obtained. The test was conducted on samples without notches.

## 3. Results and Discussion

**Figure 4:**
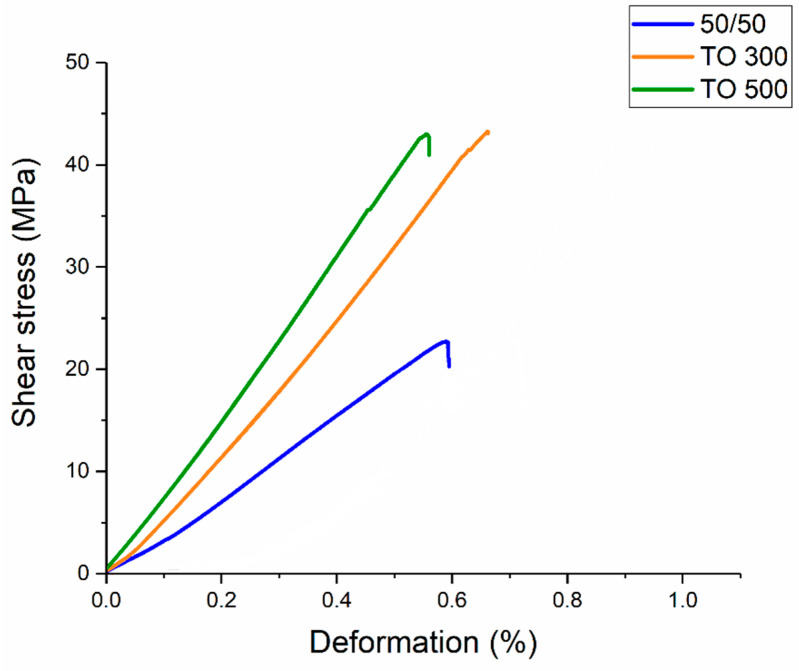
Typical load–displacement curves of composites reinforced with initial CF (50/50) and modified at different temperatures of CF (TO 300 and TO 500) during ILSS tests.

In previous studies, we investigated influence of the carbon fiber contents and surface modification conditions on the mechanical properties of PES-based composites [16]. In order to characterize the interfacial interaction between the CF and the and polymer matrix, an interlaminar shear strength (ILSS) test was conducted. Typical stress–strain curves for different types of composites are presented in Figure 4.

It was shown that the surface modification of carbon fibers allows for significantly increasing the adhesion between carbon fibers and polyethersulfone, which leads to a jump in interlaminar shear strength. As shown in previous studies, this is due to the formation of new functional groups on the surface of the fibers. Thus, shear strength rises from ~21 MPa for composites reinforced with initial fibers to ~44 MPa for materials reinforced with modified ones.

Apart from that, a strong dependence between carbon fiber content and interlaminar shear strength of the composites was found. The downward trend in shear strength magnitude with the increase in fiber content was explained by means of SEM, which showed a lack of impregnation of the carbon fibers due to insufficient volume of the polymer and, consequently, a worse stress distribution through the composites’ volume during the tests [16].

Considering the influence of surface modification of CFs on mechanical properties and the connection between shear strength and other types of mechanical loading, we found that the same patterns are observed in the three-point bending tests. The results of ILSS and three-point bending tests are presented in Table 1.

**Figure 5:**
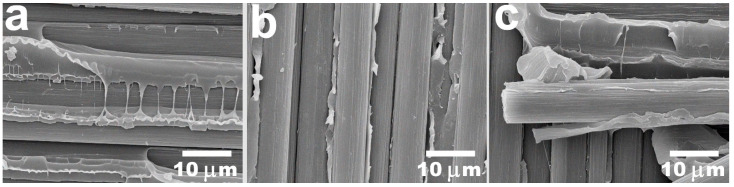
SEM images of the PES-based 50/50 (**a**), TO300 (**b**), and TO500 (**c**) composites after interlaminar shear strength tests.

The mechanical properties of the PES-based composites, collected in Table 1, show that while flexural strength has the same dependence between fiber content and surface modification, E-modulus behaves in a different way. It can be clearly seen that flexural strength and E-modulus can vary significantly depending on the composition of CRFP and the surface modification of the fibers. Thus, an increase in carbon fiber content led to worse flexural strength, while the elastic modulus experienced an upward trend. The reason for this is the fact that flexural strength more depends on the ability of the matrix to evenly distribute external load through the reinforcement, while the E-modulus is more dependent on the E-modulus of the reinforcing material itself. Consequently, while compositions with higher fiber contents had a higher elastic modulus, insufficient impregnation led to lower magnitudes of flexural strength. On the other hand, surface modification of carbon fibers allowed for increased flexural strength of the composites as well as the E-modulus. It was shown that maximum values of flexural strength of 960 MPa were recorded in the composites reinforced with fibers modified at 500 °C, while the E-modulus for the composites was at about 57 GPa. We assumed that the surface modification of carbon fibers improved both the ILSS and mechanical properties of the composites during bending due to increased interfacial interaction between the carbon fibers and PES after thermal oxidation of the CF, which was proved by scanning electron microscopy of the samples after ILSS tests. SEM images of the carbon fiber-reinforced PES-based composites after the ILSS tests are presented in Figure 5.

The SEM images proved that the main reason behind the increase in the mechanical properties of the investigated composite materials is better interfacial interaction between polymer and CFs in the composites reinforced with thermally treated carbon fibers. Figure 5a illustrates the typical fracture surface of the composites reinforced with initial CFs with a fiber content of 50 wt.%. It can be seen that polymer interlayers do not adhere to the fibers; consequently, there were a lot of gaps and cavities at the interface between the fibers and PES. In the composites reinforced with fibers after surface modification, which are presented in Figure 5b,c, another structural behavior occurs. The polymer interacts with the fibers very efficiently as it sticks to CFs. Apart from that, there are a lot of cleavages of the matrix that also indicate the tendency of the polymer to deform rather than delaminate through the CF-PES interface, which is a sequence of improved adhesion. According to the results, we concluded that the surface modification of the carbon fibers using the thermal treatment method can be an effective way to enhance the adhesion and mechanical properties of carbon fiber-reinforced PES-based composites.

**Figure 6:**
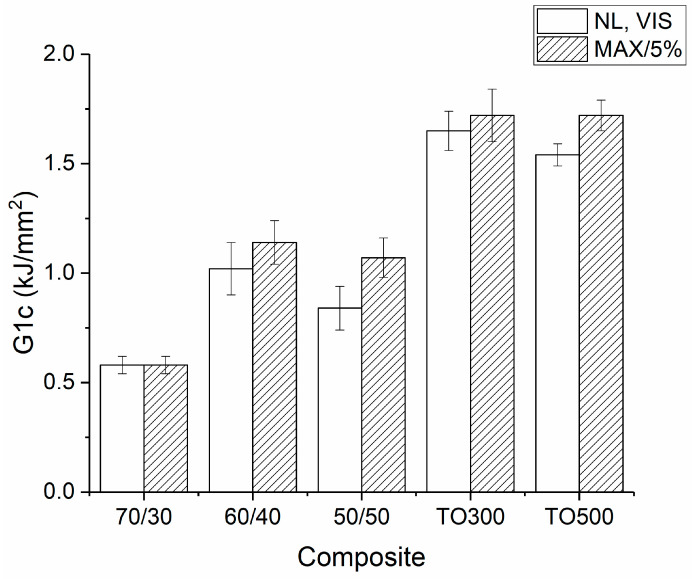
Results of the interlaminar fracture test of the PES-based composites with different fiber content and composites reinforced with modified CFs.

Thus, it was shown that the standard mechanical properties of obtained PES-based composites were strongly dependent on the fiber content in the composites. Moreover, surface modification of the carbon fibers allowed for increased interfacial interaction between the polymer and reinforcement and, consequently, the interlaminar shear strength and flexural strength of the obtained CFRP. Therefore, we assumed that interlaminar fracture toughness should be subject to the same patterns. Figure 6 depicts the results of the ILFT of the obtained composites with different fiber-to-polymer ratios and various conditions of thermal oxidation. Each value of G_1c_ was calculated with the point at which non-linearity started on the stress–strain curve or with the point of visual start of propagation of a fracture (NL, VIS) and maximum load on an initial curve (MAX).

It was shown that interlaminar shear strength is strongly influenced by fiber content and surface modification conditions of the CFs. Thus, G_1C_ reached 1.72 kJ/m^2^ for samples reinforced with carbon fibers thermally treated at 300 °C and 500 °C. On the other hand, the 50/50 sample, which had the same fiber content but was reinforced with the initial carbon fibers, had a G_1C_ value equal to 1.07 kJ/m^2^, which is 40% lower. Generally, it can be noted, that surface modification allowed for an increase in the crack resistance of composite materials due to the increased interfacial interaction between CFs and PES. The values obtained with the starting point visual propagation are often considered more conservative when compared with results gained with the point of maximum load. However, in this case, error gaps were big enough to neglect the difference between these two methods of interpreting results. The analysis of the influence of the carbon fiber content on the fracture toughness of the composites showed that the following patterns can be found. The samples with fiber contents of 70 wt.% (70/30) of CFs have the lowest G_1C_ criteria of 0.58 kJ/m^2^. The reason for such behavior could be an insufficient quantity of the polymer matrix, which results in a smaller overall surface of interfacial interaction, and the fracture propagates without the need for higher energies. Moreover, it was noted that it is impossible to distinguish among the moments when the non-linear behavior of the load–displacement curve starts, when the visual propagation of a crack starts, and the peak of the load. At the same time, the composites reinforced with 60 wt.% of carbon fibers showed a G_1c_ equal to 1.14 kJ/mm^2^, which is almost double the value compared with the 70/30 composites. The composites with 50wt.% of carbon fibers recorded G_1C_ values of 1.07 kJ/mm^2^. Hashemi et al. reported that unidirectional polyethersulfone composites have a fracture toughness of up to 0.80 kJ/mm^2^ [36]. We consider that such a gap occurs due to different geometries of reinforcement. Generally, the obtained materials showed the same patterns in behavior as those observed during the interlaminar shear strength tests, which proves the overall dependence between these two properties.

At the same time, analysis of effective E-modules, calculated using Formula (3), showed that the correlation between the modulus obtained during flexural tests and calculated during ILFT tests is not fulfilled for all composite materials. The results of the calculation of the effective E-modules for the PES-based composites are shown in Table 2.

**Figure 7:**
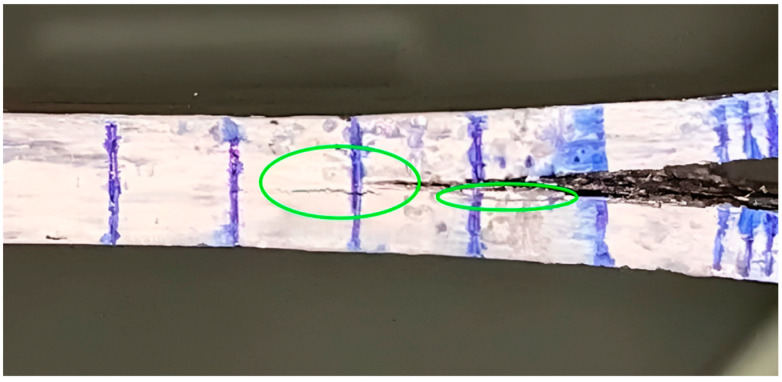
Typical view of a delaminated sample during the double cantilever beam test (50/50 sample). Green circles highlight areas of delamination in different plane from the initial one.

Particularly, only the 70/30 and TO500 composites showed a good correlation between the flexural modulus and calculated modulus values. In the other cases, the effective E-modulus tends to have much higher magnitudes compared with the flexural modulus, which is due to a much bigger value of Δ, as far as it is used for the calculation via Formula (3). The value of Δ depends on the change in compliance with the further propagation of a crack [37]. Hence, a bigger magnitude of this value shows that compliance drops slower with propagation than expected for the ideal delamination case. We consider that such a phenomenon occurs as the result of delamination in interlaminates other than the initial one, leading to a higher consumption of elastic energy and overstated values of the applied load and, consequently, fracture toughness G_1C_. Figure 7 depicts a typical view of delamination during the test. The green circles highlight areas where delamination and propagation of the crack different from the initial plane occur.

The composite with a fiber content of 70 wt.% performed in a more stable way during the tests because of poor interfacial interaction, making crack propagation the easiest way to absorb the energy. On the other hand, the TO500 sample had no delamination in other layers because of an overall better volume stability, which is the consequence of a high interfacial interaction between the fibers and the PES matrix. It is noteworthy that TO500 composites showed high stability of properties in previous research [16].

**Figure 8:**
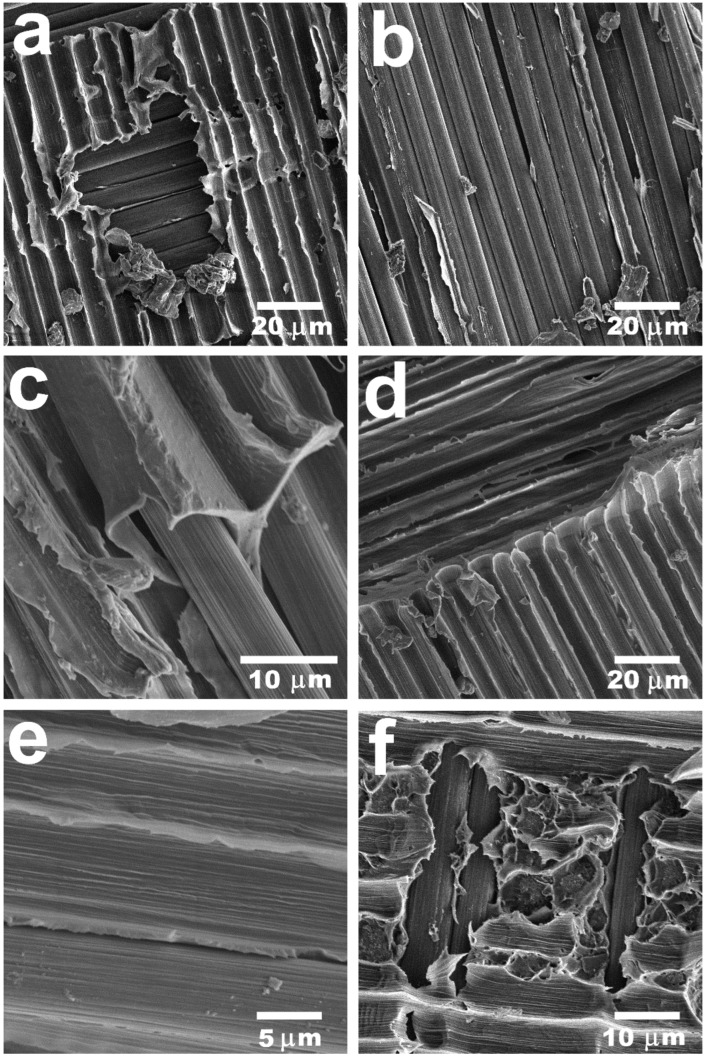
SEM images of surfaces of the PES-based composites with a fiber content of 70 wt.% (**a**,**b**) and 50 wt.% (**c**,**d**) and those reinforced with modified fibers (TO500) (**e**,**f**) after delamination.

In order to better understand the mechanisms of fracturing in composite materials, scanning electron microscopy of the samples’ surfaces after delamination was conducted. The results are presented in Figure 8. SEM images allowed us to identify interesting peculiarities in the behavior of the composites during ILFT tests. Thus, it was found that the surface of the composites with a fiber content of 70 wt.% after delamination was poorly impregnated, which can be clearly seen in Figure 8a,b. The rare areas of the remaining polymer were covered with footprints of fibers, which indicated poor adhesion between the polymer matrix and CFs, as far as there was no evidence of matrix cleavage [38]. In Figure 8c,d, the 50/50 sample is depicted. It is noteworthy that there were a lot of residual polymers left on the surface. However, because of the absence of irregularities in polymer layers and a huge number of voids on the fiber–polymer interface, it can be stated that the interfacial interaction was still insufficient. On the surface of the TO500 samples, a lot of irregularities and ruptures in the polymer layer were found. These ruptures are signs of plastic deformation that occur for many different composite systems in the case of good interlaminar fracture toughness [39]. This also can be proof of the good adhesion between the modified CFs and polymer matrix. Moreover, some fibers were still left in their initial positions (Figure 8e) despite the huge mechanical stress applied. It is also noteworthy that the SEM photos of composites after the ILSS tests (Figure 5) also showed cleavages of the matrix in the composites reinforced with modified carbon fibers. Consequently, it can be concluded that insufficient impregnation and poor interfacial interaction between carbon fibers and polyethersulfone can serve as the main reasons for the poor crack resistance of the obtained composites. Moreover, it is noteworthy that no broken fibers were found during fractography. This can be explained by lower fiber bridging during tests, as far as such phenomena normally are accompanied by a high amount of fiber debris [36]. Since composites were reinforced with twill fabrics, we assume that fibers located perpendicular to the direction of crack propagation stop fibers from bridging.

The polyethersulfone/continuous carbon fiber composite system has not been investigated widely. Apart from the quasistatic mechanical properties, dynamic ones are also of great interest. Consequently, a complex investigation of the properties of composites is an important task that is necessary to understand the fields of application of the developed materials and can lead to new peculiar behaviors of the properties of studied composite materials Moreover, it is considered that the quasistatic properties of the materials are in a dependence with dynamical properties; however, such dependence is not always directly proportional. Thus, an investigation of the impact properties of the composite materials was conducted, the results of which are presented in Table 3.

**Figure 9:**
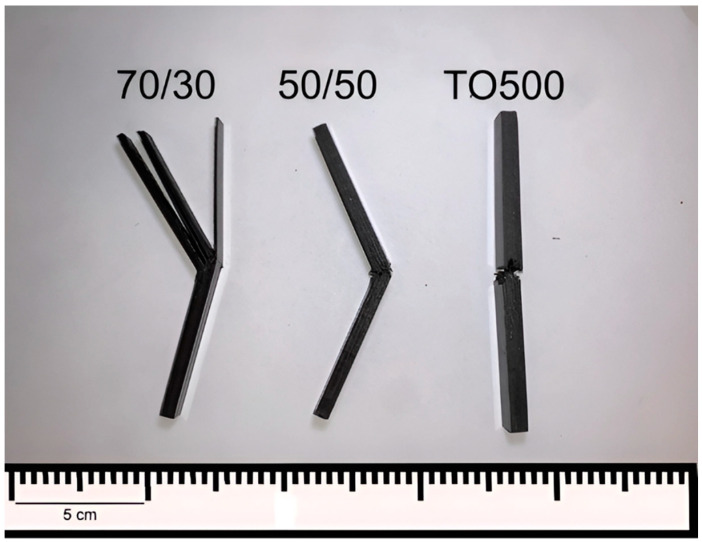
Typical types of destruction of the PES-based composites during impact strength tests.

It was shown that the composites reinforced with initial fibers have an impact strength of 155 kJ/m^2^ for composites with 70 wt.% of fiber content and 130 kJ/m^2^ for 50 wt.% compared with 86 kJ/m^2^ and 80 kJ/m^2^ of the composites reinforced with modified fibers TO300 and TO500, respectively. Such behavior can be interpreted as abnormal, considering the better mechanical properties and fracture toughness of CFRP reinforced with treated CFs. However, this phenomenon totally coincides with the observed properties. Thus, Rodgers et al. [40] showed that despite the fact the composites reinforced with modified carbon fibers needed higher kinetic energy to cause any significant damage during ballistic tests, once this limit energy was reached, composites were destructed in a brittle manner, consequently absorbing much less energy compared with composites reinforced with initial fibers. The same results were presented in several studies where an inverse dependence between interfacial bonding strength and impact properties of CFRPs based on thermoset [41] and thermoplastic [30,31] polymers was found. To explain such behavior, it is necessary to consider the character of destruction during impact. In Figure 9, the PES-based composite samples after the impact strength tests are shown.

It was found that the composites reinforced with initial CFs at the fiber content of 70 and 60 wt.% (70/30 and 60/40 compositions, respectively) were destroyed with huge delamination. Therefore, the energy of the pendulum is transferred to the energy of delamination, which occurs in multiple layers, increasing the recorded impact toughness values of the composites. On the contrary, samples reinforced with modified carbon fibers were destroyed simultaneously, without huge absorption of the kinetic energy of the pendulum. As shown earlier, the surface modification of carbon fibers allowed for a significantly increased interlaminar shear strength, which is an indicator of interfacial interaction between fibers and the matrix in composites. Thus, we consider that simultaneous destruction is a consequence of increased adhesion and interlaminar fracture toughness. Absorbing the impact energy by delamination, matrix cracking, fiber breakage, and fiber pull-out, which are more pronounced in the case of lower interfacial interaction, results in various characteristics of the destruction of the initial and modified fiber-reinforced composites. In our previous research, it was also shown that reinforcing composites with modified fibers allowed for reducing the inner free volume of composites because of better impregnation of the carbon fibers and improved adhesion of the polymer matrix. This in turn allowed for minimizing the movements of fibers during dynamic loads, which also resulted in a lower delamination and, consequently, energy absorption during impact tests [16]. Moreover, this conclusion can be also proved by standard error deviations, which are much higher for the 70/30 and 60/40 samples compared with the composites reinforced with modified CFs.

## 4. Conclusions

Interlaminar fracture toughness and impact strength of polyethersulfone-based composite materials reinforced with carbon twill fabrics were investigated. A correlation between interlaminar shear strength and interlaminar fracture toughness was found. For the study of interlaminar fracture toughness, a double cantilever beam test and modified beam theory as the method of data reduction were used. The impact strength was studied using pendulum impact on an unnotched sample.

The following conclusions can be drawn from this study:

- Thermal treatment of carbon fibers allowed for a significant increase in the interlaminar shear strength and interlaminar fracture toughness of composite materials.
- Interlaminar fracture toughness was ~1.7 kJ/m^2^ for composites reinforced with carbon fibers thermally treated at 500 °C, while composites reinforced with initial fibers showed only ~1.0 kJ/m^2^.
- Fractography by means of SEM showed that the main reasons for the lower fracture toughness of the composites reinforced with initial fibers were insufficient impregnation (for 70/30 and 60/40 compositions) and bad interfacial interaction.
- Thermal treatment negatively influenced the impact strength of composites.
- The impact strength of the composites reinforced with initial fibers was equal to ~130 kJ/m^2^, while for the composites reinforced with modified fibers, it was only ~80 kJ/m^2^.
- Such behavior occurred because of better interfacial interaction between the phases of the composites, which made delamination and fracture propagation during impact energetically insufficient compared with transverse simultaneous destruction.

## Figures and Tables

**Table 1:** Results of interlaminar shear strength and flexural tests of the carbon fiber-reinforced PES-based composites [16].

Composite	Interlaminar Shear Strength, MPa	Flexural Strength, MPa	E-Modulus, GPa
70/30	14 ± 1	482 ± 28	66 ± 5
60/40	18 ± 1	540 ± 30	58 ± 3
50/50	21 ± 1	602 ± 23	50 ± 4
TO300	42 ± 2	902 ± 26	58 ± 3
TO500	44 ± 7	963 ± 26	57 ± 2

**Table 2:** Effective E-modulus calculated after ILFT tests for the carbon fiber reinforced PES-based composites.

Composite	E_1f_, GPa
70/30	62 ± 7
60/40	80 ± 18
50/50	79 ± 23
TO300	81 ± 17
TO500	50 ± 6

**Table 3:** Results of impact strength tests of the carbon fiber-reinforced PES-based composites.

Composite	Impact Strength, kJ/m^2^
70/30	155 ± 18
60/40	157 ± 30
50/50	130 ± 5
TO300	86 ± 2
TO500	80 ± 4

## Data Availability

The data presented in this study are available on request from the corresponding author in accordance with established practice and University requirements.
